# Disease Avoidance Model Explains the Acceptance of Cohabitation With Bats During the COVID-19 Pandemic

**DOI:** 10.3389/fpsyg.2021.635874

**Published:** 2021-07-16

**Authors:** Piia Lundberg, Ann Ojala, Kati M. Suominen, Thomas Lilley, Annukka Vainio

**Affiliations:** ^1^BatLab Finland, Zoology Unit, Finnish Museum of Natural History, University of Helsinki, Helsinki, Finland; ^2^Natural Resources Institute Finland (Luke), Helsinki, Finland; ^3^Helsinki Institute of Sustainability Science (HELSUS), Department of Forest Sciences, Faculty of Agriculture and Forestry, University of Helsinki, Helsinki, Finland

**Keywords:** bats, disease avoidance, COVID-19, cohabitation, conservation, mediation, knowledge, negative emotions

## Abstract

Bats and humans have a close relationship based on cohabitation, with bats taking roost in buildings. It has been suggested that bats function as a reservoir of the SARS-CoV-2 virus that causes the COVID-19 disease in humans. A misconception that bats can spread SARS-CoV-2 to humans may have increased negative emotions toward bats and reduced individuals’ acceptance of cohabitation with bats during the COVID-19 pandemic. By applying the disease avoidance model, we tested whether knowledge about bats would be associated with reduced negative emotions toward bats, which in turn would be associated with increased acceptance of cohabitation with bats. Moreover, we tested whether previous experiences of bats, perceived COVID-19 risk, age, gender and level of education would be associated with negative emotions and acceptance of bats. A quantitative survey (*N* = 577) collected during the COVID-19 pandemic in Finland was analyzed with multiple linear regression. The results supported the disease avoidance model. Negative emotions toward bats reduced the acceptance of cohabitation with bats. However, knowledge about bats was associated with increased acceptance of bats both directly, as well as indirectly, *via* reduced negative emotions. Moreover, perceived COVID-19 risk was associated with increased negative emotions toward bats, and reduced acceptance of bats. Females were more likely than other respondents to report negative emotions, and reduced acceptance of cohabitation with bats. Prior experience of bats was associated with increased acceptance of bats as neighbors. These findings suggest that COVID-19 pandemic may threaten the existence of bats if no action is taken. The findings highlight the importance of correcting misunderstandings about non-human species as transmitters of diseases to humans.

## Introduction

Bats and humans have had a lengthy coexistence that dates back to the time when hominids began to regularly use caves (Usinger, 1966; Rossina, 2006). This is exemplified by the fact that bats and humans share their respective lineages of *Cimex lectularius*, the bed bug, which colonized humans in the shared living quarters (Roth et al., 2019). The close relationship between bats and humans, based on cohabitation, has carried to this day, with bats taking roost in buildings (Marnell and Presetnik, 2010). At northerly latitudes, bats depend on the shelter and warmth of buildings during pregnancy and lactation (Humphrey, 1975). Bats also benefit humans as they provide a wide range of valuable ecosystem services, such as pollination (Howell and Roth, 1981) and pest control (Boyles et al., 2011; Kemp et al., 2019; Weier et al., 2019). Just lately, it has been estimated that bats save vineyards with up to 7% of their production through pest suppression (Rodríguez-San Pedro et al., 2020). However, despite their usefulness for humans, bats have suffered from fear, hostility as well as cultural prejudice throughout history (Kingston, 2015). Even though attitudes toward historically stigmatized species have improved in modern times (George et al., 2016), bats are a species group still heavily associated with fear and phobias (Knight, 2008).

Bats are also associated with potentially zoonotic pathogens, including bacteria (Veikkolainen et al., 2014; Hokynar et al., 2016; Lilley et al., 2017) and viruses (Li et al., 2005; Jakava-Viljanen et al., 2010; Drexler et al., 2012). The current COVID-19 pandemic has now led to an even stronger association between bats and zoonotic pathogens than before the pandemic (Cerri et al., 2020; WHO, 2020), contributing to a more widespread view on bat persecution (MacFarlane and Rocha, 2020; Sasse and Gramza, 2020). During the COVID-19 pandemic, bats have been wrongly implied as the natural reservoir for the SARS-COV-2 virus, which causes the disease in humans (Zhang et al., 2020). Although bats provide important ecosystem services, the media in general is inclined to portray a negative image of bats; most often as reservoirs of deadly viruses (López-Baucells et al., 2018). This could be partly due to misinformation related to COVID-19 that people read and share especially on social media platforms (Pennycook et al., 2020).

The prevailing COVID-19 pandemic may affect willingness of humans to accept non-human species in their immediate environment, which may be particularly pronounced for those species with some link to the SARS-CoV-2 virus. Especially during the early stage of the pandemic, rumors circulated about the connection between COVID-19 and wildlife, however, there is still uncertainty as to which species the virus originally transferred to humans from (Neupane, 2020). The framing of the message on the origin of the SARS-COV-2 virus can have a significant influence on the opinions of people (Bolsen and Palm, 2020; MacFarlane and Rocha, 2020), and thus affect the extent to which people are willing to coexist with bats. A great deal of ambiguities and even misinformation are related to the COVID-19 outbreak (MacFarlane and Rocha, 2020; Bolsen and Palm, 2020), which can foster false beliefs of the role of bats in the disease outbreak. At least among humans, cues of disease may lead to discrimination of other human beings, including even those who do not increase the risk of disease to others (Park et al., 2003). Similarly, for bats, this could mean discriminating against them on the basis of false beliefs, which could lead to an increase in reluctance to co-exist with them. Because attitudes toward bats and perception of threats to bats are the most important factors supporting management (Fagan et al., 2018), the recent worldwide events have the potential to initiate devastating impacts on bat conservation. Therefore, there is a direct need to gain a deeper understanding on how the COVID-19 pandemic has affected attitudes toward bats.

The disease avoidance model helps to explain the process of avoidance of potential or real threat. The model is based on the assumption that humans have evolved a tendency to avoid potential threat of diseases, and this is one of the reasons for the avoidance of certain animals from which humans could contract a contagious disease (i.e., associated risk of contamination) (Prokop and Fančovičová, 2013; Hunt et al., 2017). Disease avoidance is associated with the emotions of fear and disgust toward disease- or threat-related animals (Oaten et al., 2009; Prokop and Fančovičová, 2013). These negative emotions have an adaptive function, their evolutionary role is to protect the organism from contact with contaminants and prevent the acquisition of disease (Matchett and Davey, 1991), as well as to avoid diseases (Curtis et al., 2011). People can also differ in terms of their sensitivity to have such negative emotions, and that can affect their mental health, as well as their behavior in a positive or negative direction. For example, higher disgust sensitivity could lead to high health anxiety and further, to hypochondriasis as its extreme case (Fan and Olatunji, 2013). However, disgust sensitivity could also motivate and maintain health-safety behavior (e.g., washing hands) (Fan and Olatunji, 2013). In addition to this, for contagious diseases such as Ebola and Zika, disgust sensitivity has been found to be the main driving force behind public attitudes toward these diseases and avoiding regions where the disease is present (Kam, 2019).

The omitted disease cues (either accurate or inaccurate) can lead to stigmatization and the activation of the disease avoidance system, manifested by physical and social avoidance (Oaten et al., 2011). The influencing factors of disease avoidance are the knowledge and beliefs that are related to the possible threat of disease. Ethnic outgroups or foreigners are often blamed for outbreaks of epidemic diseases, which can trigger neglect toward these groups in the human society (e.g., Faulkner et al., 2004). However, similar processes can also be directed at animals. For example, a belief in myths together with a poor knowledge of bats has been found to be related to negative attitudes toward bats and avoidance of them among both children and adults (Prokop and Tunnicliffe, 2008; Prokop et al., 2009; Musila et al., 2018). In the case of the COVID-19 disease, it is yet unclear how the (false) beliefs on the role of bats in the disease outbreak affect acceptance of cohabitation with bats. As we already know that negative verbal information can increase fear of animals (Kingston, 2015), news coverage related to zoonoses can further skew people’s attitudes toward bats (Schneeberger and Voigt, 2015).

People’s perceptions of bats may also differ by gender. For example, Musila et al. (2018) found that women had more negative attitudes toward bats and they also believed more in myths about bats than men. Similar gender differences have been observed also in other studies for animals associated with fear or disgust: attitudes of females have been more negative than those of males (Davey et al., 1998; Bjerke and Østdahl, 2004). Higher age, instead, has had both a positive (Musila et al., 2018) and a negative association (Bjerke and Østdahl, 2004) with attitudes toward bats. Higher level of education has been found to be associated with more positive attitudes toward bats (Bjerke and Østdahl, 2004; Musila et al., 2018).

Another factor that may contribute to avoiding the potential threat of disease is concern related to the disease itself. With regards to COVID-19, Shook et al. (2020) found that concern toward this disease was related to behavior that sought to prevent the onset of the disease among the United States citizens. They also found that disgust toward pathogens was related to a greater concern about the COVID-19 disease. However, sometimes concern toward the disease can also lead to irrational behavior, for instance, due to prejudices. Park et al. (2003) found that people who are more concerned about a disease may avoid contacts with disabled people, although these situations are not associated with the risk of contracting a disease. Bearing this in mind, concern toward the COVID-19 disease could influence reluctance to accept bats in the immediate environment.

The aim of this study was to explore whether the COVID-19 pandemic has affected individuals’ acceptance of cohabitation with bats. Disease avoidance model suggests that explicit knowledge, beliefs and myths that associate neutral object with disease contamination make the neutral object appear disgusting (Oaten et al., 2009, 2011; Prokop et al., 2009). As a consequence, people want to avoid that object in order to avoid contamination. Therefore, disease avoidance model suggests that the association between knowledge and object is strongly mediated by negative emotions related to disease avoidance such as disgust, fear, and perceived anxiety (e.g., Matchett and Davey, 1991; Curtis et al., 2011; Davey, 2011; Prokop and Fančovičová, 2013).

In this study we tested three hypotheses related to the disease avoidance. More specifically, we wanted to test the hypothesis that negative emotions toward bats would be associated with reduced acceptance of cohabitation with bats (Hypothesis 1). In addition, we wanted to test the hypothesis that knowledge about bats would be associated with increased acceptance of cohabitation with bats directly, as well as indirectly, *via* reduced negative emotions toward bats (Hypothesis 2). In other words, this hypothesis suggests that negative emotions related to disease avoidance would mediate the association between knowledge about bats, and acceptance of cohabitation with bats. Moreover, we wanted to test the hypothesis that increased concern about the COVID-19 risk in Finland would increase negative emotions and reduce acceptance of bats as neighbors (Hypothesis 3).

## Materials and Methods

### Survey Design and Participants

We conducted an online survey in Finland during May-June 2020. The survey was administered in Finnish and it included several questions related to opinions and knowledge of bats and COVID-19, as well as questions on respondents’ socio-demographic background. We used relevant sections of that broader survey in this study. The link to the survey was distributed in social media through institutional (University of Helsinki, BatLab Finland, Helsus, Luomus), Natural Resources Institute Finland (Luke) and personal Facebook and Twitter accounts. However, to include individuals explicitly with more experiences with bats in our sample set, a direct survey link was also sent to the participants of an ongoing citizen science (CS) “Papanapankki” project on bats. The CS project involved people who had a bat colony in either the building they live in, a building on their premises, or at their summer cottage. The CS-participants collected bat fecal pellets for researchers to determine the distribution and changes in diet composition in latitudinal and temporal gradients of different bat species. Altogether, we received 577 responses to our survey. The majority of the respondents were female (68.8%), their mean age was 45 years (SD = 14.47, range 20-91), they were highly educated and most of them lived in a city (see Table 1). Roughly one-tenth of them were participants of the CS project. In addition, more than half of the respondents (56.5%) reported to have bats in their immediate environments either in the attic of their home, courtyard building or summer cottage (Table 1).

**TABLE 1:** Sociodemographic background of the respondents (*n* = 577) and prior experiences with bats.

Variable		%	*n*
Gender^a^	Female	68.8	397
	Male	25.1	145
	Gender unknown	6.1	35
Residential area type	City	61.4	353
	Suburb	18.4	106
	Countryside	20.2	116
	Missing	0.003	2
CS-project participant	Yes	11.9	69
Level of education	Comprehensive school	1.7	10
	1–3-y. vocational degree	9.9	57
	Upper secondary school degree	10.2	59
	An engineering-, business and administration or nursing degree	5.7	33
	Polytechnic degree	14.4	83
	Lower academic degree	12.3	71
	Master’s degree or specialist medical doctor degree	32.8	189
	Licentiate or Ph.D. degree	11.1	64
	I do not want to say/missing	1.9	11
Bats occur in the immediate environment^b^	Yes	56.5	326

*^*a*^Variable gender had four categories (female, male, other and prefer not to say). The category “Gender unknown” includes the last two categories and missing values.*

*^*b*^Whether bats are present at least in one of the following places in the vicinity of the respondent: in the attic of the home, in the courtyard building or in the summer cottage. This variable reflects previous experiences with bats.*

### Measures

#### Acceptance of Cohabitation With Bats

We asked whether the respondents would accept cohabitation with bats by asking them to choose the option that corresponded to their opinion about the following statements using a 7-point Likert scale ranging from 1 = completely disagree to 7 = completely agree: I do not mind if bats use (a) my residential building as a day roost, (b) my courtyard building as a day roost, and (c) my summer cottage as a day roost. If the respondents did not have the above-mentioned premises, we asked them to imagine they would and respond on that basis. The mean score of the three items was used *(Cronbach alpha* = *0.91).*

#### Knowledge of Bats

We measured knowledge of bats using seven statements that were either true or false [e.g., Humans can get rabies from a bat bite (true)]. Respondents expressed their responses using a 5-point Likert scale. We reverse coded three items so that larger value indicated better knowledge and then calculated the mean of the items. According to Stadler et al. (2021), knowledge is formed by aspects that are not necessarily associated with each other, and therefore the Cronbach alpha should not be used for assessing how well a certain scale can measure respondents’ knowledge on a specific domain. Therefore, Cronbach alpha was not applicable for this scale. The mean variable was used in the regression analysis.

#### Negative Emotions Toward Bats

We asked the respondents to indicate their emotions of disgust, fear and anxiety toward bats at the moment using a 7-point Likert scale ranging from 1 = not at all to 7 = extremely. We calculated a mean variable from these three items for analysis (Cronbach alpha = 0.83).

#### Perceived Risk of COVID-19

We asked the respondents to express their concern about the COVID-19 situation using three questions: concern about the COVID-19 situation in Finland, the perceived risk to themselves and for people close to them. We used a 7-point Likert scale ranging from 1 = not concerned at all to 7 = extremely concerned. We calculated a mean variable from these three items (Cronbach alpha = 0.78).

#### Prior Experiences of Bats

We asked our respondents whether there are bats in their immediate vicinity in the following buildings: attic of the home, courtyard building, or their summer cottage. From these, we formed a new dichotomous variable (“prior experiences with bats”) based on the respondents’ “yes” answers (1 = there are bats in at least one of these buildings, 0 = bats are not present in any of these, Cronbach alpha = 0.90).

#### Sociodemographic Variables

We asked the respondent’s age, gender and level of education. From these, we formed a dichotomous variable “gender female” (1 = yes, 0 = no) and highly educated (1 = yes, 0 = no). We used age as a continuous variable in the regression analyzes.

Descriptive statistics of the main variables are shown in Table 2. Acceptance of cohabitation with bats correlated negatively with negative emotions and perceived risk of COVID-19 as well as with age and gender (being female). There was a positive correlation between acceptance of bats, knowledge and previous experiences with bats. Furthermore, negative emotions correlated positively with perceived risk of COVID-19 and education as well as negatively with knowledge.

**TABLE 2:** Means, standard deviations and correlations (Spearman rho).

	*M*	SD	Age	Gender (female)	Level of educ. (acad.)	Knowledge of bats	Negative emotions	Experiences of bats	Perceiv. COVID-19 risk	Acceptance of cohab. bats
Age	45.01	14.47	1.00	−0.01	−0.06	0.10*	−0.04	0.17***	0.25***	−0.10*
Gender (female)	–	–	−0.01	1.00	0.03	0.06	0.05	0.04	0.03	−0.15***
Level of educ. (acad.)	–	–	−0.06	0.03	1.00	0.11**	0.11*	0.00	−0.01	−0.05
Knowledge of bats	4.03	0.46	0.10*	0.06	0.11**	1.00	−0.28***	0.06	0.09*	0.24***
Negative emotions	1.85	1.30	−0.04	0.05	0.11*	−0.28***	1.00	0.01	0.11**	−0.48***
Experiences of bats	–	–	0.17***	0.04	0.00	0.06	0.01	1.00	0.05	0.08*
Perceiv. COVID-19 risk	4.13	1.32	0.25***	0.03	−0.01	0.09*	0.11**	0.05	1.00	−0.09*
Acceptance of cohab. bats	5.44	1.87	−0.10*	−0.15***	−0.05	0.24***	−0.48***	0.08*	−0.09*	1.00

*p < 0.05, **p < 0.01, and ***p < 0.001.

### Data Analysis

The mediation model was tested with multiple linear regression using the PROCESS v3.3 package (Hayes, 2018) and the IBM SPSS Statistics version 25.0. Knowledge was used as an explanatory variable and the acceptance of cohabitation with bats was used as the dependent variable. Negative emotions were used as a mediator variable. In addition, prior experiences of bats, perceived COVID-19 risk, age, gender and the level of education were used as covariates in the model. Indirect association between knowledge of bats and acceptance of cohabitation with bats was estimated using the 95% confidence intervals and 5,000 bootstrap samples.

## Results

The mediation model included two linear regressions (Agler and De Boeck, 2017; Hayes, 2018). They together explained about 49% of the variation in the responses, which can be considered good (Table 3). According to the results, negative emotions were associated with reduced acceptance of cohabitation with bats, as expected (Hypothesis 1).

**TABLE 3:** Results of the mediation model: unstandardized regression coefficients (*B*) and standard errors (SE).

	Model 1		Model 2	
	Negative emotions toward bats		Acceptance of cohabitation with bats	
	*B*	*SE*	*B*	*SE*
Intercept	4.57***	0.60	5.81***	0.83
Negative emotions toward bats			−0.73***	0.07
Knowledge about bats	−0.96***	0.14	0.46*	0.20
Perceived COVID-19 risk	0.14**	0.04	−0.02	0.06
Experiences of bats	0.03	0.11	0.45**	0.14
Age	0.00	0.00	−0.02**	0.01
Gender (female)	0.26*	0.11	−0.47**	0.14
Level of education (academic degree)	0.28*	0.12	0.02	0.12
*R*^2^	0.14***		0.35***	

*p < 0.05, **p < 0.01, and ***p < 0.001.

Knowledge about bats was directly associated with increased acceptance of cohabitation with bats. In addition, bootstrapped 95% confidence interval for the indirect association between knowledge and acceptance of bats was statistically significant (*B* = 0.69, *S.E.* = 0.13, *LL* = 46, *UL* = 0.96). Therefore, the association between knowledge and acceptance of cohabitation with bats was partially mediated by negative emotions, as expected (Hypothesis 2). In addition, increased perceived COVID-19 risk was associated with increased negative emotions toward bats, and reduced acceptance of cohabitation with bats as neighbors, as expected (Hypothesis 3).

In addition, females and those with an academic degree were more likely to report negative emotions toward bats than other respondents (Table 3). Older respondents and females were less likely to accept cohabitation with bats than other respondents.

## Discussion

The results suggest that negative emotions of disgust, fear, and anxiety toward bats reduced acceptance of cohabitation with bats. However, knowledge of bats was associated with increased acceptance of cohabitation with bats both directly, as well as indirectly, *via* reduced negative emotions toward bats. Moreover, increased perceived COVID-19 risk was associated with negative emotions toward bats. These results bear resemblance to the Prokop et al. (2009) study, in which a belief in myths about bats was associated with avoidance of bats. In addition, our results support the proposition of Shook et al. (2020) that increased concern of COVID-19 may lead to increased pathogen disgust sensitivity. Therefore, our study supports the disease avoidance model indicating that negative emotions related to disease avoidance such as disgust, fear and anxiety are essential emotions that mediate the association between the perceived threat object and avoidance behavior. However, no longitudinal data is yet available on the COVID-19 situation to test the causal directions between risk perception, knowledge, negative emotions and acceptance/avoidance.

It is clear that we should not underestimate the emotions that are evoked by false beliefs and myths (e.g., Onyishi et al., 2021). Emotional dispositions toward wildlife can be innate, conditioned, or consciously learned (Jakobs and Vaske, 2019). Some animals, such as spiders or snakes, evoke more innate emotional responses, explained by evolutionary threat reaction. For example, the predisposition to develop fear toward spiders and snakes is found even with 6-months-old infants by studying their pupillary reactions compared to other animals and natural objects (Hoehl et al., 2017). Such innate emotional reactions are difficult to change, especially by just giving cognitive information. However, communication to change learned beliefs is possible (Jakobs and Vaske, 2019), yet difficult if these beliefs and myths are loaded by conflicting or misleading information and shared by many people (Castillo-Huitrón et al., 2020).

In this day and age, information travels rapidly through various social media channels, which also applies to information on COVID-19. People may not, however, be critical enough of the material shared on social media and therefore they may continue to endorse and share false beliefs related to the ongoing pandemic (Pennycook et al., 2020). These misconceptions and false beliefs, in turn, can affect attitudes toward species associated with spreading the disease and be detrimental to their conservation. From the disease avoidance perspective, it has been suggested that the association between the disgust elicitor, negative emotions such as disgust and avoidance behavior is rather automatic (Oaten et al., 2009). Therefore, interventions aiming at reducing “false alarms” may be more successful if they focus on correcting false beliefs instead of reducing negative emotions related to disease avoidance. However, correcting false beliefs may be challenging because people tend to selectively pay attention to information that confirms their prior beliefs and behaviors (Vainio, 2019). Therefore, our results further underline the importance of explicitly correcting these false beliefs on the role of species in the pandemic. For example, even in scientific literature on virology, bats have often been wrongly portrayed as the cause of diseases that threaten humans (López-Baucells et al., 2018), which may further negatively impact their status even among the scientific community. Therefore, instead, the role of bats as providers of ecosystem services (e.g., their benefits to humanity) should be emphasized. So far, this emphasis has been seriously lacking in scientific debate, especially in the field of virological research (López-Baucells et al., 2018).

We also found that females, more likely than other respondents, reported negative emotions (fear, disgust and anxiety) toward bats and less acceptance of cohabitation with bats. Our results mirror findings on gender differences from previous literature, in which females have shown more negative attitudes toward bats (Prokop et al., 2009; Musila et al., 2018), as well as other species associated with fears (Røskaft et al., 2003; Prokop and Fančovičová, 2010). The same phenomenon was also observed in disgust-relevant animals in a cross-cultural study: females expressed more fear toward disgust-relevant species than males, reflecting the greater disgust sensitivity in females (Davey et al., 1998).

Higher age was associated with lower acceptance of cohabitation with bats in our study. In accordance, younger people showed greater tolerance for snakes in Greece compared to older people (Liordos et al., 2018). When studying human preferences for a variety of species, Bjerke and Østdahl (2004) also found that age was negatively associated with preferences for bats, corresponding to the decreased willingness to cohabit with bats observed in our study. However, higher age has also been observed to have a positive association with attitudes toward bats (Musila et al., 2018). Because of these inconclusive results it is evident that more studies are needed to clarify the role of age.

It is good to bear in mind that negative views on bats have neither been universal nor always prevailed in the human history. For example, in the Asia-Pacific region, bats have been associated with good luck (Rocha et al., 2021) and for the most part attitudes toward bats have been positive in that area (Low et al., 2021). However, global pandemics can exacerbate human attitudes toward bats. For instance, a study in China showed that the COVID-19 outbreak had changed people’s attitudes toward bats to a more negative direction, mainly due to misconception on the relationship between bats and COVID-19. Even the specially organized bat conservation lecture failed to correct the misconception that bats transmit SARS-CoV-2 to humans directly. The authors suggested that this was due to the frequent inaccurate media coverage, general cultural bias, but also the way virologists talk about the associations between bats and diseases (Lu et al., 2021).

Global pandemics and the linking of diseases to bats can also increase support for bat culling. In many parts of the world, bats have been persecuted as a consequence of their role as the probable origin of SARS-CoV-2. For instance, there were multiple reports of bats evicted from houses after the beginning of the pandemic in China, some of which led to direct deaths of bats (Zhao, 2020). Some countries, such as Indonesia, adopted bat culling as a strategy to combat COVID-19 (CMS, 2020; Tsang, 2020). With science linking bats to COVID-19, the public and policy managers may have directly or indirectly related bats to COVID-19, which has led to the repelling and culling of bats. However, these misunderstandings may drive new threats to bats (MacFarlane and Rocha, 2020; Zhao, 2020). For now, there is no evidence that culling bats is an effective measure to control bat-borne diseases (Hallam and Mccracken, 2011; Streicker et al., 2012), and furthermore, culling may in fact increase the spread of bat-borne viruses and risk to humans (Plowright et al., 2008; Amman et al., 2014; Plowright et al., 2015; Olival, 2016).

Traditionally, bats have been regarded as an object of respect in Nordic countries (Eklöf and Rydell, 2021). For example, in a review of old literature in Sweden and the Swedish-speaking parts of Finland, Eklöf and Rydell (2021) found no support for bats being considered dangerous or pathogenic among Nordic people in the past. Indeed, the general attitude toward bats in Finland, where our study was conducted may be very different to that in countries where cullings have taken place today. Historically, bats were considered powerful creatures in Nordic countries. They were well known and highly respected in terms of amulets of good luck and ingredients of magical charms (Eklöf and Rydell, 2021). A bat colony in a house was, in fact, believed to protect the inhabitants from any illnesses in Finland (Wessman, 1952). These ancient beliefs may still be reflected in the results of our study. The Christian church introduced bats as evil creatures in the middle ages, but old beliefs were not discarded, and pagan beliefs and Christianity flourished side by side (Eklöf and Rydell, 2021). The behavior and nocturnal activity of bats was associated with some dark activities, but never in the Nordic history were they considered to carry any illnesses or diseases (Eklöf and Rydell, 2021). Besides this, bats and their roosts are strictly protected by law in Finland, which would ultimately prevent any culling practices from taking place.

### Limitations of This Study

The analysis was based on a convenience sample conducted through the Internet and so the results cannot be generalized to the whole population. The link to the survey was distributed in social media both through authors’ institutions as well as personal accounts, and therefore it is possible that respondents are more interested in nature compared to the general population. In addition, a part of the participants was recruited from the bat related CS project. In general, the participants were rather amiable toward bats, as the estimated negative emotions toward them were rather low. Furthermore, the knowledge of bats was relatively good among the respondents and there were clearly more people in our sample who accepted bats as their neighbors compared to the ones who did not. However, the purpose of the study was not to obtain a representative view of the distribution of knowledge of bats, negative emotions and acceptance of bats in Finland, but to test the associations between these constructs. The mediation model explained the total variance in the data well, and the Cronbach alphas were good (>0.70) for all mean variables. Moreover, our study was based on the analysis of cross-sectional data, and therefore, the directionality of the associations between knowledge, negative emotions, acceptance of cohabitation with bats and perceived risk of COVID-19 cannot be tested. Thus, additional studies with longitudinal datasets are needed to explore the causal directions between these variables.

However, for novel diseases such as COVID-19, information on their possible impact on attitudes toward species, such as bats, is still limited, and therefore our study provides a new insight on this topical issue. Furthermore, our study provides novel information on the perceptions of adults about bats since previous research on attitudes toward bats has often been conducted from the perspective of children or university students (e.g., Knight, 2008; Prokop and Tunnicliffe, 2008; Prokop et al., 2009; Borgi and Cirulli, 2015).

### Final Consideration

In our view, our study increases the understanding of the willingness of people to cohabit with wildlife in the COVID-19 -landscape present in 2020, at least from the viewpoint of those individuals who followed social media channels and answered our questionnaire. This information is important for the conservation of wildlife, as conservation measures need support from humans, and pandemics such as COVID-19, can affect whether people accept wildlife in their immediate environment. Social media channels are also important in this regard—the information that spreads rapidly through them can affect people’s opinions, whether it is real information or based on misunderstandings. Because of the increasing encroachment of natural habitats, the coexistence of humans and wildlife is being put under more and more pressure.

In particular, the protection of species that live in close vicinity with humans requires coordinated cooperation between stakeholders to put bat-related health risks into context and to provide society with understanding of the importance of our coexistence with wildlife and the environment in general (Rocha et al., 2020). Awareness could be promoted through integrating nature-city-interactions, to assist in understanding the human-animal-environment nexus from a “shared risk” perspective (Vanhove et al., 2020). This kind of integrative approaches, that jointly consider the health of humans, animals and the environment have been adopted by approaches such as One Health, EcoHealth, and Planetary Health.

Public awareness and understanding are a necessity to provide effective conservation measures to ensure the viability of biodiversity and the important ecosystem services (Rocha et al., 2020). Often knowledge alone is not enough to enhance positive attitudes, but changes are more associated with increasing knowledge through a subjective experience. If the public is unwilling to cohabitate with some species due to false beliefs (e.g., a belief that bats are reservoirs of diseases such as COVID-19), these experiences are not sufficient. As an example, CS combines research, education and civic participation, and can be a useful link in this regard. By participating in citizen science projects, people gain environmental knowledge and science interpretation skills, but also facilitate the collection of large datasets that are out of the reach of researchers alone (e.g., Krasny and Bonney, 2005; Jordan et al., 2011). This win-win concept has the potential for large-scale societal changes in attitudes and enhancing criticism toward the media in public. Therefore, stakeholders should better acknowledge and utilize this method in future endeavors to steer the relationship between humans, wildlife and the surrounding environment in a more positive direction.

## Data Availability Statement

The datasets presented in this article are not readily available because the survey was conducted in Finnish, and the data of this project will be stored in the Finnish Social Science Data archive in 10 years after this project has been ended under the project: Naapuruussuhteen rakentaminen lepakoihin kansalaistieteen voimin (English, Building a neighborly relationship with bats through citizen science). Requests to access the datasets should be directed to https://www.fsd.tuni.fi/en.

## Ethics Statement

Ethical review and approval was not required for the study on human participants in accordance with the local legislation and institutional requirements. Written informed consent for participation was not required for this study in accordance with the national legislation and the institutional requirements.

## Author Contributions

PL, AO, AV, and TL contributed to conceptualization and funding acquisition. PL, AO, and AV contributed to investigation. AV analyzed the data. AO supervised the project. All authors contributed to methodology and writing the original draft.

## Conflict of Interest

The authors declare that the research was conducted in the absence of any commercial or financial relationships that could be construed as a potential conflict of interest.
